# Efficient Prediction of Co-Complexed Proteins Based on Coevolution

**DOI:** 10.1371/journal.pone.0048728

**Published:** 2012-11-09

**Authors:** Damien M. de Vienne, Jérôme Azé

**Affiliations:** 1 Bioinformatics and Genomics Programme, Centre for Genomic Regulation, Barcelona, Spain; 2 Universitat Pompeu Fabra, Barcelona, Spain; 3 UMR CNRS 8623, Laboratoire de Recherche en Informatique, Université Paris-Sud, Orsay, France; Technical University of Denmark, Denmark

## Abstract

The prediction of the network of protein-protein interactions (PPI) of an organism is crucial for the understanding of biological processes and for the development of new drugs. Machine learning methods have been successfully applied to the prediction of PPI in yeast by the integration of multiple direct and indirect biological data sources. However, experimental data are not available for most organisms. We propose here an ensemble machine learning approach for the prediction of PPI that depends solely on features independent from experimental data. We developed new estimators of the coevolution between proteins and combined them in an ensemble learning procedure.

We applied this method to a dataset of known co-complexed proteins in *Escherichia coli* and compared it to previously published methods. We show that our method allows prediction of PPI with an unprecedented precision of 95.5% for the first 200 sorted pairs of proteins compared to 28.5% on the same dataset with the previous best method.

A close inspection of the best predicted pairs allowed us to detect new or recently discovered interactions between chemotactic components, the flagellar apparatus and RNA polymerase complexes in *E. coli.*

## Introduction

Protein-protein interactions are involved in most cellular processes. The knowledge of the complete network of protein interactions of a given organism (its “interactome”) helps to understand complex biological processes such as signalling cascades, metabolism or transcription control [1]. It is also useful for assigning functions to unknown proteins, based on the function of their interacting partners.

Proteins that interact, whether physically or not, are expected to be co-evolving: any evolutionary event affecting one protein might indirectly or directly impact all of its interacting partners (reviewed in [2]). This hypothesis is at the basis of a number of computational methods aimed at systematically predicting functional associations between proteins, i.e. proteins belonging to a cellular complex.

The Phylogenetic Profiles method (PP, [3]) explores the patterns of presence/absence of proteins in a set of related species: it assumes that if two proteins interact to perform a given function, the loss of one of them is followed by the loss of the other one, leading to similar Phylogenetic Profiles. Genomic Context (GC, [4]), looks at the conservation of the gene neighbourhoods in different species: the physical proximity of two genes is expected to be conserved among species if their products interact. Two other methods require multiple sequence alignments to be performed. These are the *in silico* two-hybrid method (I2H, [5]), where correlated mutations between proteins are estimated from multiple alignments of orthologous sequences, and the widely used *mirrortree* method [6] where phylogenetic trees are indirectly compared by estimating the correlation between the pairwise distance matrices computed from a multiple sequence alignment. Trees with high similarity (high correlation coefficient between distance matrices) are expected to represent interacting protein pairs while trees with low similarity (low correlation coefficient between distance matrices) represent non-interacting proteins.

In recent years, machine learning approaches have been applied to protein interaction detection, especially for the identification of protein-protein interactions in yeast. These new methods are based on the integration of data from multiple heterogeneous sources, including experimental ones: protein sequences, protein interactions derived from high-throughput experiments, gene expression data, Gene Ontology terms, co-regulation data, localization data, mRNA expression fluctuations during the yeast life-cycle, essentiality data, etc. [7]–[11]. These methods proved to be very efficient even with a limited number of features considered [9]. However, because these methods require diverse and sometimes numerous experimental data, their use was restricted to *S. cerevisiae*, as these data are not available for most other organisms.

Recently, García-Jiménez *et al.* [12] proposed a new learning method for the detection of PPI based on combined data from various prediction methods developed independently (PP, GC, I2H, *mirrortree* and Gene Fusion (GF)). Even though this new combined approach, applied to *E. coli*, gave better results than each prediction method taken independently, the number of False Positives (FP) and False Negatives (FN) remained worth considering. Moreover, because different prediction methods call for different types of data and independent implementation, the computation of all of the features is time-consuming and missing values are frequent.

In this study, we propose a new approach for detecting proteins belonging to complexes involved in specific cellular functions. Apart from the generation of a learning set (a gold-standard dataset), this method does not rely on experimental data, but solely on genome sequences. It extracts features related to coevolution between proteins and uses a machine learning approach to combine them. We developed new features based on two published methods, namely PP and *mirrortree*. Improved version of the basic Phylogenetic Profiles method have already been proposed (for example, see [13]). Here we improved it by computing *quality measures* inspired by data mining methods, taking into account the number of species where a given protein is present, the size of the overlap between the set of species where orthologs of the proteins are found and the maximum number of species (i.e. the number of genomes studied). Concerning the *mirrotree* method, many improvements have been proposed: removal of the background similarity between the trees prior to the *mirrortree* analysis (*tol-mirror*, [1], [14]), use of the complete coevolutionary network (*context-mirror*, [15]), restriction of the *mirrortree* method to conserved regions in the protein domain sequences [16] or supervised learning using the phylogenetic species tree [17]. In our approach, we developed features based on the topological comparison between the proteins trees in addition to the comparison of their distance matrices as is normally done. It is indeed accepted that a phylogenetic distance matrix does not completely reflect the topology of a tree, leading to the loss of potentially important information. Finally, since the comparison between the individual protein trees and the species tree is crucial, we also estimated the topological similarity and the similarity based on distance matrices between each protein tree and the Tree of Life (ToL).

To challenge the efficiency of our method, we used it to detect PPI in *E. coli* since we have a good dataset of its interactome. We then compared our set of predictions to those obtained using the *mirrortree, tol-mirror* and *context-mirror* methods with the same *E. coli* dataset.

We show that new features, directly inspired from old ones based on coevolution, associated with a powerful combination of classifiers in a learning procedure, allowed the prediction, with an unprecedented precision, of the interactions between proteins in organisms for which experimental data are not available. We obtained an area under the ROC curve (AUC) of 0.93 with our method, a value surpassing that of the *context-mirror* method [15] (AUC = 0.87). Further, we designed a filtering method to remove negative pairs in order to increase the ratio of positive over negative examples. Such a filtering procedure resulted in a very clean dataset free of almost all negative examples but still containing half of the positive ones.

Finally, we analysed in detail the 50 best predicted pairs. We focused on 3 well-known complexes: chemotactic components, the flagellar apparatus and RNA polymerase complexes in *E. coli*, allowing detection of new links between them by previously unpublished interactions. Most of these new links are concordant with text-mining results and one of them is even confirmed by a binding experiment performed after the creation of the gold standard dataset used here. This demonstrates the validity of our method and gives new insights on a complex self-assembling nanomachine that allows bacteria to move in their environment and swim up chemical gradients.

## Results

### Prediction of interacting pairs

We present the results in terms of ROC curve, AUC, recall and precision curves, using (i) different methods proposed earlier to predict protein-protein interaction: *mirrortree* [6], *tol-mirror* [1], [14] and *context-mirror* [15] and (ii) our method, using the same dataset in every case, obtained as described in Material and Methods.

**Figure 1:**
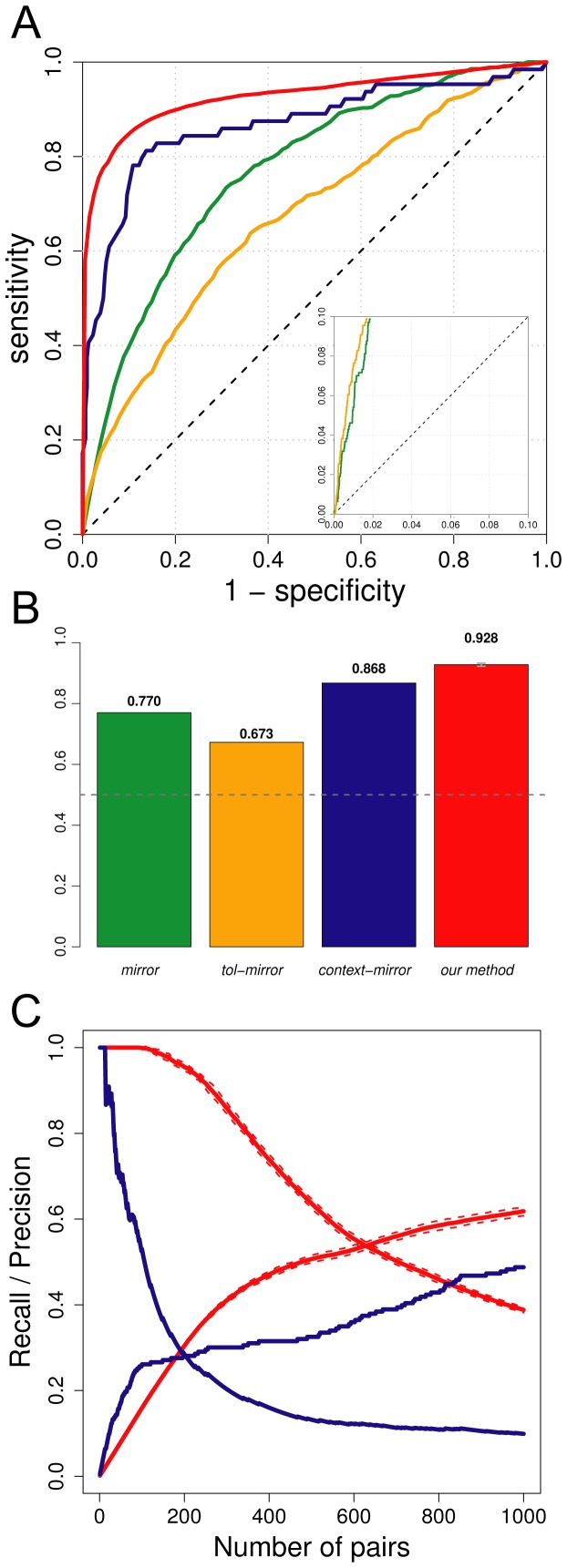
Evaluation of the efficiency of the PPI detection method proposed here. **A.** ROC curves of the four methods compared. Green: *mirror*; Orange: *tol-mirror*; Blue: *context-mirror*; Red: our method. The ROC curve plotted for our method is the mean of all 30 independent ROC curves obtained. A zoom of the beginning of the ROC curve is provided in the right-bottom corner. **B.** AUC values for the four methods compared. Numbers on top of each bar are the exact AUC values. The dashed gray line represents the expected AUC for a method not doing better than random. The error on the last bar represents the standard deviation over the 30 repetitions of our method. Colours are the same as in A. **C.** Comparison of the precision and recall curves for the *context-mirror* method (blue lines) and the method we propose here (red lines). The dashed lines on the curves for our method represent the standard deviation over the 30 repetitions. Only the first 1000 pairs are represented.

First, the *mirrortree* and *tol-mirror* approaches seem to perform poorly compared to the recent *context-mirror* method and to the method we propose here. This is clear from the ROC curves presented in Figure 1A and the AUC values confirm this result, with 0.77 and 0.67 for *mirrortree* and *tol-mirror* methods respectively, while the *context-mirror* and our proposed method give AUCs of 0.87 and 0.93 (*sd* = 0.0028 for the latter) respectively (Fig. 1B). Surprisingly, the *mirrortree* method seemed to give better results than *tol-mirror* according to the AUC values. This is unexpected because *tol-mirror* was created to improve the predictive power of the *mirrortree* method by removing the background similarity between matrices due to speciation events. When looking at the beginning of the ROC curve however (zoom in the right-bottom corner of Fig. 1A), the *tol-mirror* appears better at the beginning of the ranking.

The precision curve allows an estimation of how good the separation between positive and negative examples is along the sorted list of pairs. Our method gives a precision of 100% for the first 90 pairs, with a standard deviation of 0 in this interval (Fig. 1C). This means that there are no mistakes in the ordering of positive and negative pairs for the first 90 pairs. For the *context-mirror* method, the precision is 100% for the 13 first pairs only, and it then declines quickly to 28.5% for the 200 first pairs. At this cut-off, our method still shows a mean precision of 95.5%. The recall curve shows how many pairs have to be explored to retrieve, for example, 50% of the positive pairs. This happens after 450 pairs with our method, while for the *context-mirror* method it requires exploring further than the 1000*^th^* pair (Fig. 1C).

### Effect of different classifiers and their combination

The combination of the 8 classifiers (JRIP, PART, J48, RF and their bagged version bJRIP, bPART, bJ48 and bRF), gave a prediction efficiency higher than each classifier taken independently (Table 1). The bagged version of the classifiers were always better than their non-bagged version, which is expected [18]. PART and its bagged version were more efficient than the other classifiers, while JRIP and bJRIP seemed to perform poorly compared to the others. When combining only the three best classifiers (bPART, bJ48 and bRF) the AUC was the same as when using the 8 classifiers, but the precision and recall curves in this case were lower, emphasizing the fact that the AUC only gives a global, and thus not accurate, vision of the classification efficiency of a method. Overall, combining the information from different classifiers was beneficial. This is because different classifiers have different biases, and their effect could be minimised by combining the classifiers.

**Table 1:** Comparison of the 8 classifiers and of their combination.

	Non-bagged classifiers				
	*JRIP*	*PART*	*J48*	*RF*	
AUC (mean)	0.67	0.86	0.8	0.83	
AUC (sd)	0.0131	0.015	0.024	0.0081	

	Bagged classifiers				
	*bJRIP*	*bPART*	*bJ48*	*bRF*	*ALL*
AUC (mean)	0.79	0.92	0.9	0.91	0.93
AUC (sd)	0.0083	0.0048	0.0062	0.0028	0.0028

### Contribution of the different classes of features

We investigated the ability of each of the 4 classes of features (*topology, matrix, tree* and *PP*, see Material and Methods) taken independently to correctly rank the positive and negative pairs of the *E. coli* interaction dataset (Table 2).

**Table 2:** Prediction efficiency of the different classes of features.

	Classes of features				
	*topology* 1	*matrix* 2	*tree* 3	*PP* 4	*ALL* 5
AUC (mean)	0.78	0.80	0.84	0.92	0.93
AUC (sd)	0.0054	0.0045	0.0039	0.0036	0.0028

1 Features included: *I_cong_*, *I_cong_A* and *I_cong_B*.

2 Features included: *mirror*, *mirror_A_*, *mirror_B_* and *tol – mirror*.

3 Features included: *I_cong_*, *I_cong_A*, *I_cong_B*, *mirror*, *mirror_A_*, *mirror_B_* and *tol – mirror*.

4 Features included: all Phylogenetic Profile features in Table 4.

5 Features included: all features in Tables 3 and 4.

First, all classes of features are able to produce a ranking of positive and negative examples better than random (AUC>0.5). Features of the *topology* and *matrix* classes have similar prediction efficiency (AUC = 0.78 and AUC = 0.80 respectively), while their combination (the *tree class*) improves the prediction efficiency (AUC = 0.84, sd = 0.0039). This shows that the features based on tree topology and those based on matrix comparisons measure different “aspects” of the coevolution between proteins. The PP class alone is good at predicting protein-protein interactions, with an AUC of 0.92 (sd = 0.0036). The AUC is however improved when combining the *tree class* and the *PP class* of features (AUC = 0.93, sd = 0.0028, last column in Table 2). This reveals that features based on topology, matrices and PP are able to extract some independent aspects of the coevolution between proteins, their combination allowing a clear improvement of the predictive power of the method.

### Filtering out non-interacting pairs

We developed a method for filtering out non-interacting pairs, that is removing the highest proportion of negative examples but at the same time disregarding the lowest proportion of positive ones. A simple solution to this problem would be to remove a pre-determined number of pairs at the end of the ranked list of pairs. However this has two limitations: it requires an *a-priori* on the number of pairs to remove, and it does not guaranty an optimal AUC of the remaining pairs, because it does not take advantage of the prediction of each classifier taken independently. Our proposed approach uses a threshold (*α*) associated with the percentage of classifiers predicting that a pair is not interacting (see Material and Methods).

**Figure 2:**
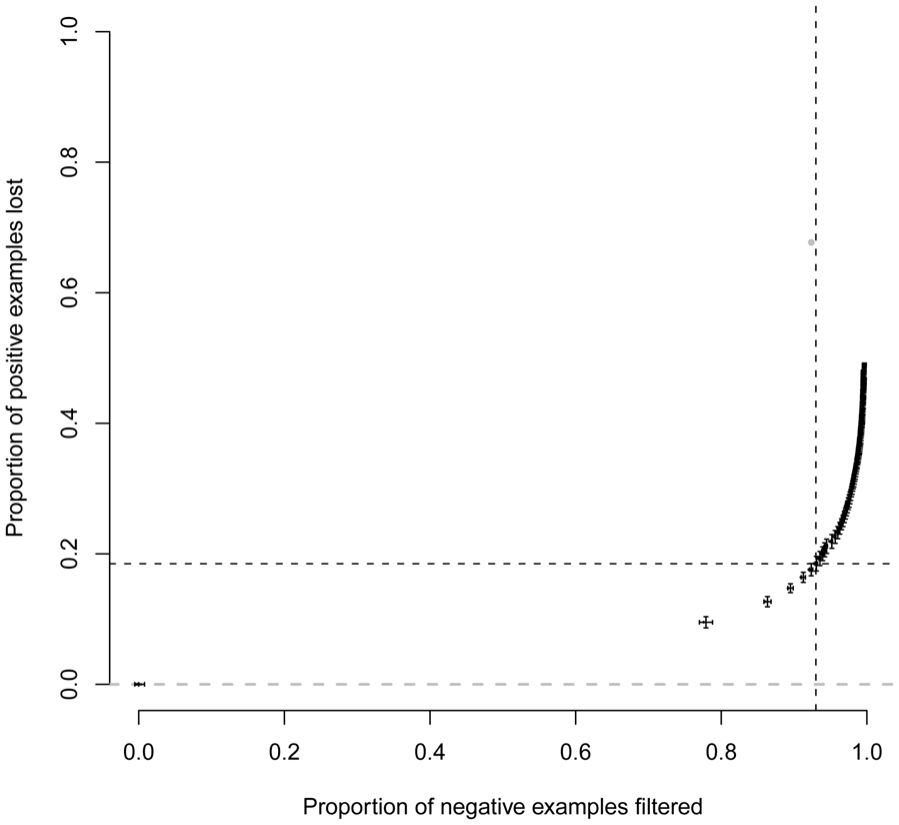
Effect of filtering out negative pairs on the proportion of positive ones lost. A perfect filtering method would produce the dashed grey line. The large grey dot represents the effect of the filter present in the *context-mirror* method. The small black dots represent what we obtain by varying the value of *α* from 0.01 (top right) to 1 (bottom left). The black dotted lines show the proportion of positive examples that are lost when our filtering method is tuned so that the proportion of negative examples filtered out is the same as the one of the *context-mirror* method. The horizontal and vertical error bars on black dots represent the standard deviation of the mean over 30 repetitions.

In order to evaluate the ability of our method to correctly filter out negative pairs, we plotted the proportion of negative examples filtered out against the proportion of positive examples lost for different values of *α*, from 0.01 (stringent filter) to 1 (no filter at all; black dots in Fig. 2). A perfect filtering method would give the pattern represented by the grey horizontal dashed line in Fig. 2: whatever the proportion of negative pairs removed, no positive pairs are lost. This would mean that the ranking of positive and negative pairs is perfect. We also represented the result of the filtering applied by the *context-mirror* method by the grey dot in Fig. 2 (93% of negative examples removed and 67.7% of positive examples disregarded). For *α* = 0.01, on average, 99.6% of the negative pairs are removed and in the same time 48.1% of the positive pairs are filtered out. So even with a very strong filter, our method is still less prone to removing positive pairs by mistake than *context-mirror*. If *α* is tuned so that the proportion of negative examples filtered out is the same as for *context-mirror* (93%, obtained for *α* = 0.93, vertical black dashed bar in Fig. 2), then the proportion of missing positive examples decreases to 18.5% (compared with 67.7% for the *context-mirror* method). Thus, our method filters out negative pairs without losing too many positive ones. The effect of the filtering strength on the quality of the ranking (AUC) for the different groups of features presented in Table 2 is provided in the next section.

### Effect of filtering on the quality of the ranking

**Figure 3:**
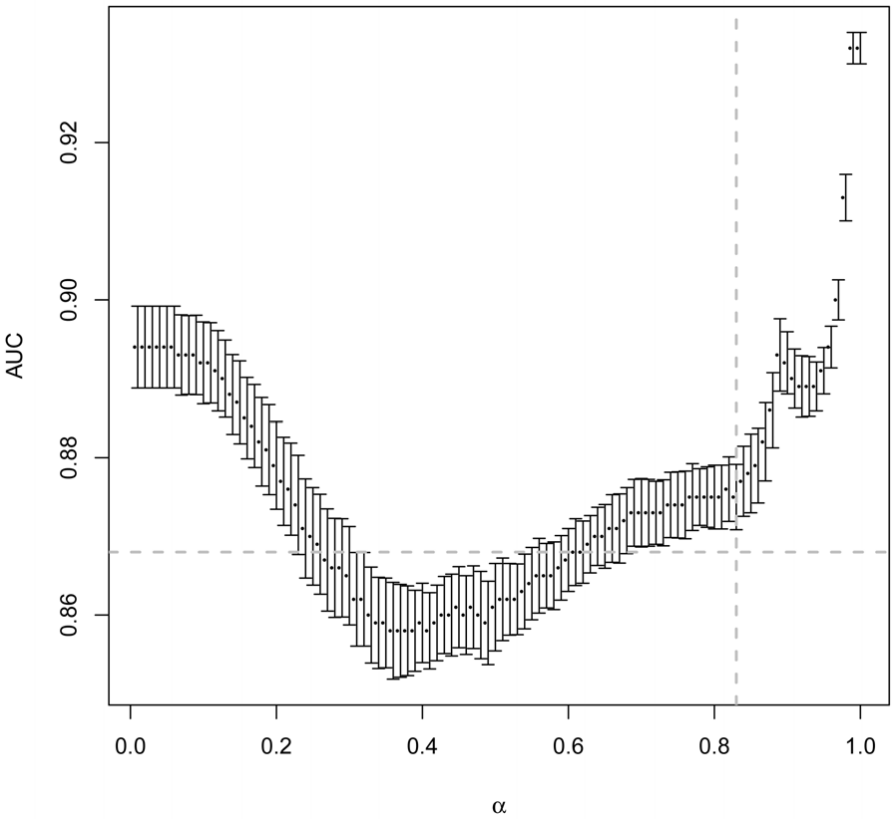
Effect of the filtering strength (*α*) on the AUC after sorting of the pairs. *α* goes from strong filtering (*α* = 0.01) to no filtering (*α* = 1). The horizontal dashed grey line represents the AUC value of the *context-mirror* method. The vertical dashed grey line represents the value of *α* for which the number of negative pairs in the dataset is the same as for *context-mirror*.

The total number of examples analysed has an impact on the evaluation of the method because it impacts the AUC values. Thus, we computed the AUC of the ranked list of protein pairs with different filtering strengths, for values of *α* between 0.01 and 1 (Figure 3). We see that for very strong or very weak filters, the AUC is always higher than the one obtained when using the *context-mirror* method (dashed horizontal grey line on Figure 3). For intermediate levels however (*α* between 0.3 and 0.6), the AUC does not appear better with our method than with *context-mirror*. This could be seen as a decrease of the efficiency of the method when a moderate filter is applied because the overall ranking of positive and negative examples is not better with our method than with *context-mirror* in this case. This shows the limitation of using AUC for estimating the quality of a method. Indeed, whatever the value of *α*, the number of positive examples analysed is always higher than with the context-mirror method (Fig. 2) and also the precision of the method is unchanged because the filter will never eliminate pairs that are ranked at the beginning of the sorted list of pairs. In other words, our method in some cases may produce more mistakes than the *context-mirror* method but for pairs of proteins that are assigned a low score and are thus ranked towards the end of the sorted list of pairs. However, the precision is always better (Figure 1C) and the number of positive examples lost is always smaller (Figure 2).

### Effect of filtering on the different groups of features

**Figure 4:**
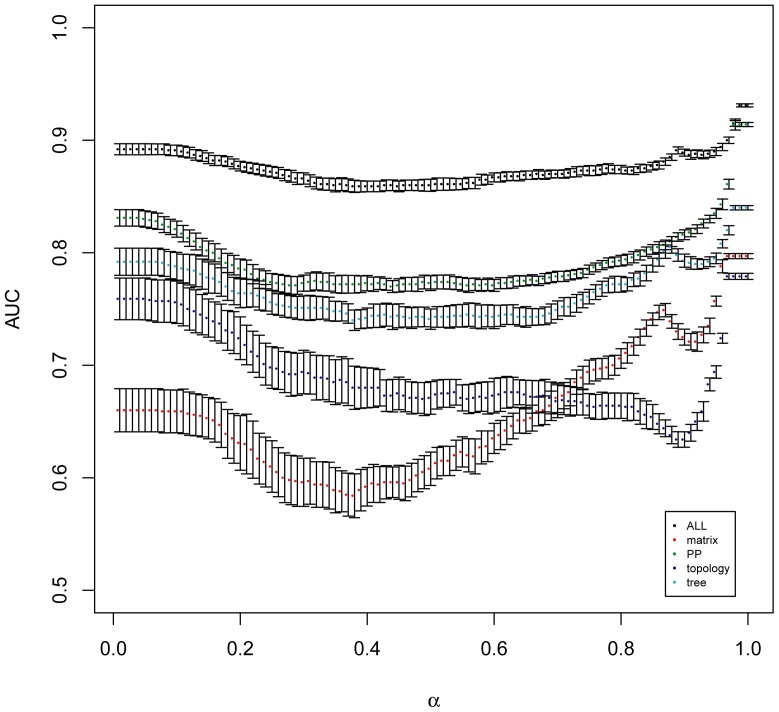
Effect of the filtering strength (*α*) on the AUC after sorting of the pairs using the different groups of features presented in Table 2. *α* goes from strong filtering (*α* = 0.01) to no filtering (*α* = 1).

We compared the effect of filtering on the AUC when using the different classes of features presented in Table 2 (Figure 4). The filtering has less impact when all of the features are combined than when only a subset of them is used. The *PP* and *tree* classes of features have similar behaviour with respect to *α*. The *matrix* class performs better than the *topology* class for high values of *α* (>0.7) but performs worse when *α* decreases. The combination of different classes of features thus results in a method whose behaviour in terms of AUC is almost uniform with respect to filtering.

### New insights into the flagellar system of E. coli

**Figure 5:**
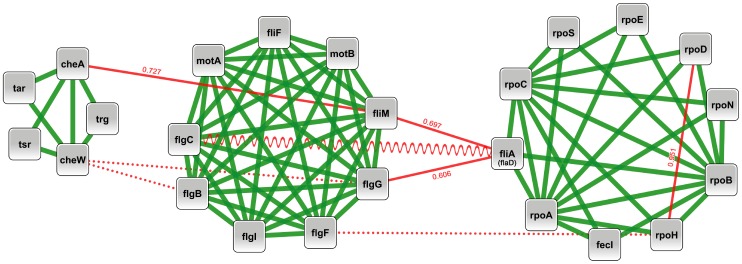
New insights into the flagellar system of *E. coli*. Chemotactic components, flagellar apparatus and RNA polymerase complexes of *E. coli*, connected by previously unknown interaction links. Green lines: previously known interaction; Red lines: previously unknown interactions; Dotted red lines: interactions for which no text-mining evidence is present in the STRING database; Wave-like red line: interaction verified by experimental work after the gold standard dataset was created [21]; plain red lines: putative interactions according to STRING text-mining data. The value associated with each solid red line is the STRING score for the text-mining evidence.

To estimate the efficiency of our approach, we focused on the nature of the pairs of proteins considered as non-interacting according to the goldstandard dataset we used (negative pairs). Among the 50 best predicted pairs, almost 50% of them (23 out of 50) are referenced in the STRING database [19] as possible interactions (Table S1). Fig. 5 illustrates the importance of discovering new links associating several clusters inside a set of protein complexes.

The DNA-directed RNA polymerase core enzyme (subunits RpoA, RpoB, and RpoC), is known to bind one of the seven sigma factors (encoded by genes *rpoD, rpoE, rpoH, rpoS, rpoN, fecI* and *fliA*, respectively) depending on physiological conditions. Unexpectedly, we detected a link between the housekeeping sigma factor RpoD (sigma 70) and the heat shock sigma factor RpoH (sigma 32). Such a link would be in accordance with the finding that the *in vitro* expression of the *rpoH* gene was found to require RpoD [20]. The flagellar sigma factor (sigma 28) FliA, is found to connect to different elements of the flagellar nanomachine. First, there is a link with the proximal rod section (FlgB, FlgC, FlgF) through the distal rod FlgG, and another link with the C-ring, FliM being one constituent of the switch complex that is essential for assembly, rotation and directional control of the torque-generating stator complex (MotA and MotB). Secondly, the link detected between FliA and FlgC (wave-like red line in Fig. 5) could be particularly important because it is strongly supported by a recent yeast-2-hybrid experiment revealing a direct interaction between these two proteins [21]. Moreover, we detected a link between RpoH and FlgF which is not published in the STRING database. Interestingly, such a link has been demonstrated in *Brucella melitensis* [22] where RpoH2 (a homologue of *E. coli* RpoH) is involved in the expression of the proximal rod protein FlgF. The two flagellar components FlgG and FliM that interact with FliA are themselves found to be connected to two chemotaxis proteins, cheW and cheA, respectively. The link between the C ring FliM and cheA could be a supplementary safety to the well known interaction between FliM and the phosphorylated form of CheY necessary to induce the switch in the clockwise/counterclockwise rotation of the flagellum. Indeed, CheY, the response regulator of bacterial chemotaxis, is phosphorylated by the histidine kinase CheA. It has been reported [23] that CheA, FliM, and CheZ display overlapping binding surfaces on CheY. Taken together, the best predicted pairs seem to be putative but plausible interactions whose further study may give new insight into protein-protein interactions in *E. coli*. More work is needed to investigate all of these pairs (listed in Table S1).

### Controlling for redundancy

We wanted to confirm that the high accuracy of prediction that we had with our method was not simply due to the dataset being highly redundant, with homologous proteins having homologous interacting partners. Thus, we predicted protein interactions using only sequence similarity and computed the ROC curve and the AUC after ranking the predicted pairs according to their e-values (see Material and Methods for details of the method used). Using only sequence similarity, we obtained an AUC between 0.52 and 0.53, depending on the method used for detecting homologous sequences.

This value is very close to what would be expected if the ranking was done randomly (AUC = 0.5). These tests show that sequence similarity alone does not provide sufficient information to predict protein-protein interactions, and consequently that the high accuracy we obtained with our proposed method cannot be accounted for by redundancy in the data.

## Discussion

We have designed a new machine learning method for the prediction of protein-protein interactions and used it to predict interacting proteins in the model organism *Escherichia coli*. Our method is conceptually based on coevolution of protein partners and uses features inspired by two methods developed earlier: *mirrortree* [6], [24] and PP [3]. First, we developed features based on the topological comparison of the protein trees in addition to the comparison of their distance matrices, as for previously proposed methods [1], [6], [14], [15], [17], [24]. We insist that distance matrices could not totally reflect their tree topology, i.e. phylogenetic relationships between the species present in the compared trees, although they contain information on the branch lengths that may be important to detect coevolution. The second type of features we developed was designed to better exploit the concept of Phylogenetic Profiles. Improvements to the initial PP method were proposed previously [13]. Here, we used *quality measures* normally used in data mining approaches in order to detect coevolution based on pattern of presence/absence of proteins in the different species.

It is difficult to estimate the independent contribution of each feature to the predictive power of a method such as the one we propose here. However, (i) the combination of features based on topology alone and matrix alone gave better prediction (higher AUC) than each group of features taken independently, proving that different aspects of coevolution between proteins can be extracted depending on the way the trees are compared. (ii) *Quality measures* contributed to increase the predictive power of the method and confirmed that extended computations based on PP could give more information than the classical PP method as initially proposed by Pellegrini *et al.* [3]. We believe that all these new features should be considered seriously in future work on PPI detection. Globally, compared to the best method proposed to date (the *context-mirror* method, [15]), our method ranks positive and negative pairs more efficiently (AUC of 0.93 compared to 0.87 with the *context-mirror* method for the same dataset) and also filters out negative pairs with more accuracy, by losing fewer positive ones.

It is important to note that the method we propose here and the three methods we compare with, including *context-mirror*, are conceptually and methodologically different. While our method is based on a machine learning approach, so that a dataset of known interactions is required for *training* the model, the other methods do not rely on such a process. Comparing the performance of machine learning methods like ours and *ab-initio* methods can still be done, but one has to keep in mind that their requirements and range of applicability are not the same.

The combination of the outputs of the 8 classifiers was made using an ensemble learning method. Each classifier is trained to predict if two proteins are interacting or not, and a degree of confidence is associated to each prediction. Ensemble learning is known to behave better than single classifiers [25], [26], and our results seems to confirm the previous results observed for ensemble learning. Moreover, and importantly, it allowed us to develop a way of filtering out negative pairs by tuning the *α* parameter. We believe that this is an important aspect of this work. Depending on whether the goal of the PPI detection is either to score the maximum number of pairs and to reconstruct the complete network of interaction, or to obtain a reduced list of pairs in which one wants to be sure of having only positive ones, the *α* parameter can be changed, from no filter (*α* = 1) to a strict filter. We see that with a very strong filter (*α* = 0.01), more than 99% of the negative pairs were removed with concomitant loss of only half of the positive ones. This means that we were able to go from a dataset including 0.817% of positive pairs to a dataset made almost exclusively of positive ones.

Reducing the size of the dataset is very important for going deeper into the detection of “direct” physical interaction between proteins. As in previous methods, our approach considers that any two proteins present in the same complex form a *positive* pair and proteins not involved in a complex are *negative* ones. Thus, our method cannot differentiate between direct and indirect physical interactions. Disentangling these two types of interaction is a difficult task that has been subject to much work in the recent years. Having reliable candidates for putative direct interactions is at the basis of a class of computational methods aimed at predicting the 3-dimensional structure of protein complexes, called “docking”. Our work could therefore be seen as a promising first step for the detection of direct interactions and the subsequent docking of proteins, by limiting the number of interactions to be tested. These methods are indeed computationally very intensive, so reducing the number of pairs that are tested is beneficial.

To check whether our method was promising we chose to study a high quality dataset of experimentally demonstrated interactions between known proteins, the *E. coli* interactome. Our method is designed to be based on coevolution, without having to deal with the incorrect assignment of each pair in each class (**pos** or **neg**). We tested it with proteins that are known to interact in functional complexes (co-complexed proteins) as opposed to proteins that belong to gene regulatory or metabolic networks. Proteins belonging to the same complex are expected to coevolve more strictly than proteins involved in the same pathway. Indeed, methods that predict PPI based on coevolution have traditionally been better in detecting co-complexed proteins than proteins sharing a pathway (see [15]), and we believe that coevolution alone is not sufficient for detecting proteins sharing the same pathway.

By focusing on the 50 first predicted pairs, we were able to propose new interactions between chemotactic, flagellar and RNA polymerase complexes. Some of these interactions were confirmed by recent experimental results and others were in accordance with results obtained in other closely related species. This confirms the validity of our approach and its ability to correctly detect co-complexed proteins. A closer look at well ranked negative protein pairs might permit in the future to gain new insights into the function of specific protein complexes whose structure and function is not yet completely understood.

## Materials and Methods

### General principle of the method

**Figure 6:**
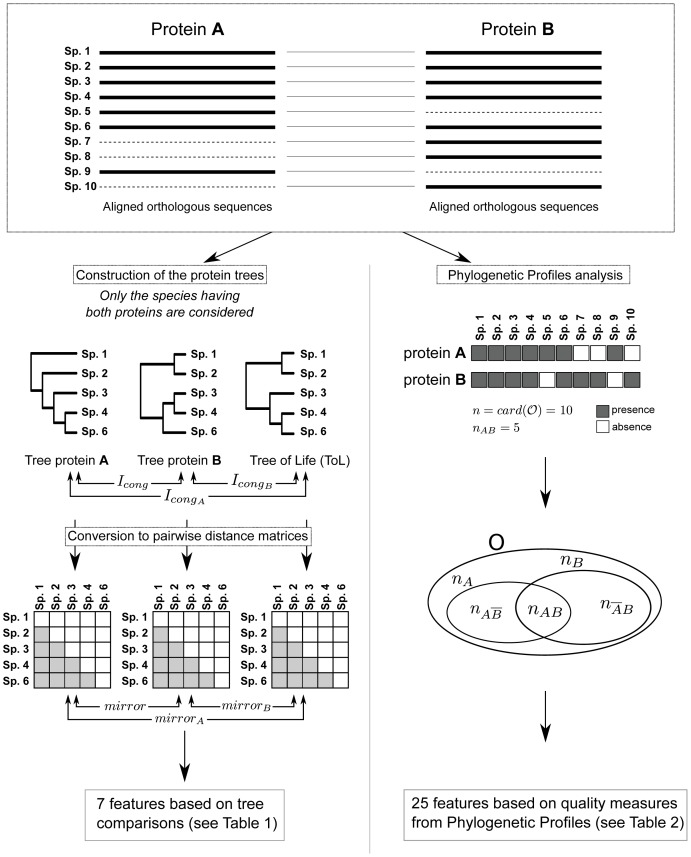
Basic description of the construction of the features based on coevolution used in this study.

For each protein in the *E. coli* genome, orthologous sequences in 115 other prokaryotic genomes were retrieved (see [15] for a list of genomes). The sequences were then aligned, leading to 2177 multiple alignments. Each protein was then compared to each other in a pairwise manner. The array of species where a given protein is present represents its phylogenetic profile. The comparison between proteins was performed either by the comparison of their phylogenetic profiles (right part in Figure 6) or by the comparison of their phylogenetic trees (left part in Figure 6). These two types of comparison led to a total of 35 features (Tables 3 and 4) used as input for a learning procedure that learns 8 classifiers and combines them in a way that allows obtaining an optimal sorting of the protein pairs. The dataset and tree reconstruction method used are provided in Material and Methods, along with the learning method. In order to estimate how effective our method was for predicting PPI and to compare it with previous methods, we sorted all the pairs according to their score (see Material and Methods) and computed on this ranked list the precision, recall and ROC curves, as well as the area under the ROC curve (AUC). The same attributes were computed using three other methods (*PP*, *mirrortree* and *context-mirror*) on the same dataset (see SI Material and Methods).

**Table 3:** List of Tree comparisons features computed in this study.

Name	Description/formula	References
*mirror*	Tree similarity between proteins A and B computed as the correlation between their pairwise distance matrices	[6], [24]
*mirror_A_*	Tree similarity between protein A and the Tree of Life (ToL) with the mirror method	[6], [24]
*mirror_B_*	Tree similarity between protein B and the ToL with the mirror method	[6], [24]
*tol – mirror*	Tree similarity between proteins A and B based on the mirror method after correction of their pairwise distance matrices to remove the background similarity due to speciation of the species themselves	[1]
*I_cong_*	Topological similarity between the trees of proteins A and B as estimated by the size of the maximum agreement subtree (MAST) between the two trees	[32]
*I_cong_A*	Topological similarity (*I_cong_* index) between the tree of protein A and the ToL	[32]
*I_cong_B*	Topological similarity (*I_cong_* index) between the tree of protein B and the ToL	[32]

**Table 4:** List of Phylogenetic Profiles features computed in this study.

Name	Description/formula	References
*n_A_*, *n_B_* and *n_AB_* confidence	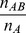	[36]
recall	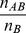	[37]
lift	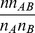	[38]
dice	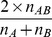	[39]
pearson	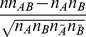	[40]
GI	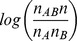	[41]
IQC	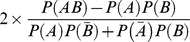	[42]
confidenceCentered 1	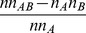	[43]
confidenceCentered 2	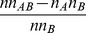	[43]
leastContradiction 1	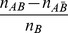	[44]
leastContradiction 2	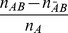	[44]
jaccard 1	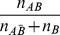	[45]
jaccard 2	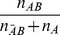	[45]
loevinger 1	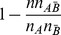	[46]
loevinger 2	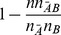	[46]
tec 1	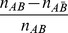	
tec 2	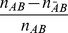	
LAP 1	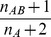	[47]
LAP 2	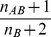	[47]
GAN 1	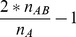	[48]
GAN 2	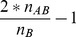	[48]
Zhang 1	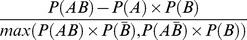	[49]
Zhang 2	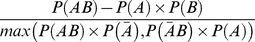	[49]
Pearl 1	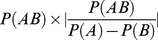	[50]
Pearl 2	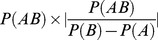	[50]

### Gold standard datasets

We applied our method to the prediction of the *E. coli* interactome. Interacting proteins in *E. coli* were retrieved from the Ecid database [27]. We focused on pairs of proteins interacting in complexes because they are expected to be more prone to coevolution than proteins present in the same pathways or proteins being co-regulated. We extracted from the Ecid database protein pairs coming from the EcoCyc database [28] and representing proteins interacting in well-known complexes whose curation had been done manually. Using this reliable dataset, we ensure a minimum amount of False Positives and False Negatives, allowing an evaluation of the quality of the method and not of the quality of the data on which it is applied.

A pair of proteins was considered positive (**pos**) if the two proteins were part of the same complex and negative otherwise (**neg**). This led to a total of 628 positive pairs (0.82% of the complete dataset) and 76 202 negative pairs (99.18% of the complete dataset), for a total of 76 830 pairs analysed.

### Tree reconstruction and comparisons

We used the same set of *E. coli* proteins as used by Juan *et al.* [15]. The authors kindly gave us the 2 177 protein sequences from the *E. coli* genome as well as their orthologous protein sequences from a set of 115 fully sequenced prokaryotic genomes (see [15] for a description of the orthologous relationships retrieval). For each one of the 2 177 groups of orthologous protein sequences, we used the program MUSCLE [29] for the multiple alignments of each protein sequence with its orthologous sequences, we cleaned the alignments by removing poorly aligned positions and divergent regions using the program Gblocks [30] and we used the program phyml [31] to recontruct the phylogenetic trees. Then for each pair of proteins, all the features presented in Tables 1 and 2 were computed. Proteins present in less than 7 species were excluded from the analysis and pairs of proteins with less than 7 species in common were not considered. This value of 7 was chosen because the *I_cong_* index used afterwards to test the topological congruence between the trees was not designed for pairs of trees with less than 7 leaves [32].

### Construction of the Tree of Life

For each of the 115 species considered in this study, the 16S rRNA sequence was retrieved from the Ribosomal Database Project (RDP) web server [33]. The MUSCLE [29], Gblocks [30] and phyml [31] programs were then used successively on these sequences in order to get the phylogenetic Tree of Life (ToL).

### Description of features based on tree comparison (tree class)

Features in this first group were computed after each multiple alignment had been converted into a phylogenetic tree (left part of Figure 6). Two features, *mirrortree* and *tol-mirror*, were computed as proposed initially by their authors. For the *mirrortree* metric, each tree was converted into a pairwise distance matrix by summing the lengths of the branches separating two leaves in the tree (i.e., patristic distance matrices). The tree similarity was then evaluated by computing the linear correlation coefficient between the two matrices. The second classical method is *tol-mirror* as proposed by Pazos *et al.* [1]: the distance matrices extracted from the trees are modified in order to remove the background similarity between the trees due to the speciation of the species themselves. We followed the protocol proposed in the original article [1], using the distance matrix extracted from the Tree of Life (ToL) to correct the protein distance matrices extracted from the protein trees. The third and fourth features, *mirror_A_* and *mirror_B_* were obtained by computing the linear correlation coefficient between each individual protein tree (after transformation into a distance matrix) and the ToL. Note that the *mirrortree* approach we used here is similar to the one used in [15] (the trees are reconstructed from the the multiple sequence alignments and are subsequently transformed into distance matrices by summing the branch length between each pair of species) and thus differs from the initial description of *mirrortree* [6] where no tree is built and the compared distance matrices are directly computed from the multiple sequence alignments. The fifth feature is the topological similarity between the protein trees compared, estimated by the *I_cong_* index [32]. This index is based on the calculation of the Maximum Agreement Subtree (MAST) metric between two trees compared. It represents the probability that the observed MAST of the two trees compared is obtained by chance alone. This measure does not take branch lengths into account but provides some information on the phylogenetic relationships between the species in the trees, information that can be partly lost by the conversion of trees into distance matrices. The two last features (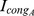 and 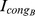) were the topological congruences between each protein tree and the Tree of Life calculated using the *I_cong_* index described previously. In total, 7 features based on the original *mirrortree* method were computed.

### Description of features based on Phylogenetic Profiles comparison (PP class)

This second group of features does not require the construction of phylogenetic trees. Instead, the phylogenetic profile of each protein is obtained by looking at the pattern of presence/absence of orthologues of each protein in the other genomes. It is usually the case that if two proteins have similar phylogenetic profiles, they are likely to be interacting. However, the number of genomes in which these proteins have an orthologous protein relative to the total number of genomes we are looking at seems extremely important. The Phylogenetic Profile problem is similar to the problem of comparing the intersection between two sets in mathematics. Consider protein family *A* is present in a set of *N_A_* species and protein family *B* is present in *N_B_* species. The idea of PP is to say that if *N_A_* and *N_B_* are identical sets, then the proteins are certainly in interaction. This is true only if *N_A_* and *N_B_* are smaller than 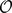, the total number of species considered (otherwise it would represents ubiquitous proteins). However, it is rare to find two protein families with exactly the same phylogenetic profile. Then different metrics exist that can estimate the degree of overlap between the two sets, taking into account the size of each set, the size of the intersection and the size of the complete set. Transposed to our problem, 25 features are computed (Table 2) representing 16 different quality measures (some of them being asymmetrical). For each pair (*A*, *B*) of proteins, these measures are calculated using as inputs: the number of species where protein *A and* protein *B* have an ortholog (*n_AB_*), the number of species where protein *A* has an ortholog but protein *B* has not (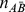), the number of species where protein *B* has an ortholog but protein *A* has not (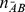) and the total number of species studied (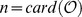, right part of Figure 6). Note that 9 of these quality measures are not symmetrical (they treat differently *A* and *B*). These measures are duplicated in order to apply them in both directions.

### Feature types and encoding

The features we used in this study are all related to coevolution and are of two types: those based on the comparison of phylogenetic trees (*tree class*, Table 3) and those based on the comparison of the Phylogenetic Profiles (*PP class*, Table 4). Figure 6 describes the way the different features were obtained from the multiple alignments of two proteins *A* and *B*. The *tree class* can be separated into two subclasses, namely the *matrix class* where the distance between the trees is computed by the linear correlation coefficient between the pairwise distance matrices extracted from the trees, and the *topology class* where topological distance between the trees is computed using the *I_cong_* index [32]. A detailed description of these features is provided in SI Material and Methods. In addition to the features based on tree comparisons and phylogenetic profile comparisons, we also included as features for each protein pair: the number of species where protein *A* has an ortholog (*n_A_*), the number of species where protein *B* has an ortholog (*n_B_*), and the number of species where protein *A* and protein *B* have one ortholog (*n_AB_*). Note that these values are those used for the computation of the features based on PP comparison (Table 4).

### Principle of the learning method

We used a 3 fold cross-validation (3CV) approach to test the ability of our method to correctly predict interacting pairs in the *E. coli* genome. The complete set of positive (**pos**) and negative (**neg**) examples (protein pairs) was separated into three groups containing each the same proportion of examples in the **pos** and **neg** classes. Two groups were used for the training part of the method, and the remaining group was used for testing. Each of the three groups is alternatively used as the test-group. This allows us to score the entire data set. This operation was repeated 30 times to ensure that the method was reproducible and thus reliable.

### Learning algorithms

We used a combination of 4 classical supervised classification algorithms to predict positive (**pos**) and negative (**neg**) classes. These algorithms are present in the most recent version of weka [34] (Weka version 3-6-4 was used in this work) and are of two types: Rules (PART and JRIP) and Decision trees (J48 and RandomForest (RF)). The bagged version of each classifier was also used (bPART, bJRIP, bJ48 and bRF) leading to a total of 8 classifiers. The combination of these classifiers is presented in the next section.

### Combination of the classifiers

Each classifier predicts, by default, the class **pos** if the probability associated with this class is greater than or equal to 0.5 and the class **neg** otherwise. Both the number of classifiers that are in agreement for assigning a specific pair to a given class and the probabilities associated with the predictions are indicators of the “confidence” that one can have in the prediction. We exploited this confidence in order to compute a global score and thus a rank associated to each example predicted.

This score is calculated as follows:
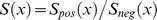
where *x* represents the example to predict and *S_pos_*(*x*) (resp. *S_neg_*(*x*)) the score associated to example *x* for the **pos** class (resp. **neg**). The calculation of the scores is detailed hereafter:
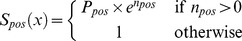


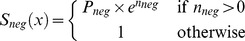


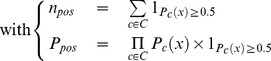


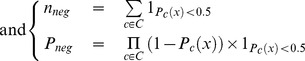
where *C* represents the set of classifiers and *P_c_*(*x*) represents the “confidence” that *x* is assigned to class **pos** by classifier *c*. *n_pos_* (resp. *n_neg_*) represents the number of classifiers that assign *x* to class **pos** (resp. **neg**) when considering 0.5 as the threshold that separate the two classes.

As a consequence, a pair for which all classifiers assign the class **pos** with a high probability will have a high score, while a pair for which all classifiers assign the class **pos** but with a low probability (only slightly higher than 0.5) will be assigned a lower score.

### Precision, Recall, ROC and AUC to evaluate the quality of the ranking

We give here a brief definition of the attributes used in this study to evaluate the quality of the ranking of positive (**pos**) and negative (**neg**) protein pairs

- **Precision** is the ratio of the number of True Positive pairs (TP) that have been retrieved to the total number of pairs predicted as positives (TP+FP). This can be computed for each cut-off in the list of ranked pairs, by considering that this cut-off represents the separation between positive and negative examples. Doing so for each possible cut-off (from pair 1 to the total number of pairs) allows a curve to be drawn representing the quality of the ranking. If the ranking is perfect, then all the interacting pairs (positive examples) will have the highest scores and thus this curve will remain at the value 1.0 until the cut-off reaches the actual number of positive pairs and decrease linearly afterwards.
- **Recall** is the ratio of the number of True Positive pairs that have been retrieved (TP) to the total number of positive pairs (TP+FN). As for the precision, the recall can be computed for each possible cut-off in the ranked list of pairs. It starts from 0 and, if the ranking is perfect, is expected to increase linearly to 1 (when the cut-off equals the actual number of positive pairs) and remain at 1 thereafter.
- **ROC** stands for the Receiver Operating Characteristic. It is a plot of the sensitivity (True Positive Rate, TPR) versus the False Positive Rate (FPR). A method able to perfectly separate positive and negative examples would lead to a ROC curve starting from position [0,0], going straight to the coordinates [0,1] along the y-axis (TPR) and then reaching the position [1,1]. Conversely, a method that would not do better than random for ordering negative and positive examples would lead to a ROC curve close to the diagonal. The Area under the ROC Curve (AUC) can also be computed. It has the value 1 for a perfect prediction and the value 0.5 for a prediction not better than random.

### Filtering out negative pairs

The machine learning method we propose here also allows filtering out the pairs that have a small score, thus enriching the final dataset in positive examples. We set a threshold for the *P_neg_* value, only for pairs s.t. *n_pos_* = 0 so that if *P_neg_*≤*α*, the pair was kept, otherwise we removed it.

Here we used *α* = 1 (no filter) for the overall evaluation of the method. We then varied the value of *α* from 0.01 to 1 in order to evaluate the effect of the strength of the filter on the trade-off between the proportion of negative examples filtered and the proportion of positive examples lost.

### Exploration of the best predicted pairs

We used the final sorted list of pairs to investigate the nature of negative pairs that were ranked highly (were assigned a high score) with our method. These represent protein pairs that have features in common with known co-complexed proteins. We focused on the 50 first predicted pairs (listed in Table S1). We used the STRING database [19] to look for possible evidence of interaction, restricting the use of STRING to text-mining evidence (proteins co-mentioned in Pubmed abstracts) and experimental evidence, because co-occurrence across genomes and neighbourhood conservation are evidence that are not independent from the phylogenetic profile used in our method.

### Comparison with other methods

Two out of the 35 features used in our approach are classical methods for detecting PPI. These are the *mirrortree* and the *tol-mirror* methods. We compared our results with those obtained using these methods independently, by comparing the ROC curves and the area under the ROC curve (AUC) after the ranking of the pairs. In addition, we compared our approach with the *context-mirror* method proposed by Juan *et al.* [15] that appears to be the best method to date for predicting PPI in *E. coli*. The principle of this method is to evaluate the similarity of each pair of protein trees in the light of the complete network of similarity between protein trees, using a linear correlation coefficient between the distance matrices extracted from the protein trees as an indicator of the similarity between trees. We used the program developed by the authors to perform this analysis, using as an input the same dataset as we used for testing our own approach. We used the default p-value threshold (*p*≤10^−5^) and considered the default levels proposed by the program: 1, 5, 10 and 25. For the AUC and ROC computations, we looked for the best *ρ* cut-off (see [15] for details) to get the maximum AUC, so that we could compare our method with the best results obtainable with the *context-mirror* method. The best AUC was obtained when filtering for *ρ*>0.6. For the *context-mirror* method, we also evaluated the quality of the ranking of the pairs by the precision and recall curves prior to the filtering based on *ρ* and compared it to the precision and recall curves with our method.

### Controlling for redundancy in the dataset

Similar (homologous) proteins might have the same protein interactors so that a redundant dataset could artificially explain a highly accurate prediction. In order to control for redundancy in the dataset, we predicted PPI using only sequence similarity. For each of the 2 177 proteins in the *E. coli* proteome, we found its best homolog (if any) in the other 2 176 proteins, using either HMMER3 (http://hmmer.janelia.org/, [35]) or BlastP (NCBI-BLAST version 2.2.26, http://blast.ncbi.nlm.nih.gov/Blast.cgi?PAGE=Proteins) with default parameters. Using a leave-one-out approach we assigned to each pair of proteins the label (positive or negative interaction) of their correponding pair of homologous proteins, if present. Finally, if p1 and p2 are two proteins and pp1 and pp2 are their corresponding homologous sequences, we assigned to the pair p1-p2 a score computed as:
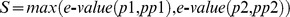
where the e-value is the sequence similarity score as returned by HMMER or BlastP. We then ranked all the pairs based on this score and computed the area under the ROC curve (AUC) for this new prediction. We expect a high value of the AUC if the dataset is highly redundant and a small value of AUC otherwise.

## Supporting Information

Table S1 **List of the 50 best ranked negative pairs detected in this study.**
(PDF)
