# Supplementary material for: Efficient Prediction of Co-Complexed Proteins Based on Coevolution
Source: PLoS One. 2012 Nov 9;7(11):e48728. doi: 10.1371/journal.pone.0048728 (PMC3494725; doi:10.1371/journal.pone.0048728)
Supplement: Table S1 — List of the 50 best ranked negative pairs detected in this study. (PDF) [file pone.0048728.s001.pdf]

# Supplementary Table S1

List of the 50 best ranked negative pairs detected in this study.

| P1 | Description | P2 | Description | Score | Evidence from STRING | STRING combined score | REMARKS |
|---|---|---|---|---|---|---|---|
| thiQ | Thiamin transporter subunit; Part of the ABC transporter complex thiBPQ involved in thiamine import, Responsible for energy coupling to the transport system (Probable) (232 aa) | modC | molybdate transporter subunit; Part of the ABC transporter complex modABC involved in molybdenum import, Responsible for energy coupling to the transport system (By similarity) (352 aa) | 366.59 | none | | Both are part of the ABC transporters |
| ilvH | acetolactate synthase III, thiamin-dependent, small subunit (163 aa) | leuC | 3-isopropylmalate dehydratase large subunit (EC-4,2,1,33); Catalyzes the isomerization between 2-isopropylmalate and 3-isopropylmalate, via the formation of 2-isopropylmaleate (466 aa) | 271.35 | Neighborhood in the Genome (homologous genes in other genomes): 0.800 Coexpression: 0.419 | 0.875 | |
| rpoH | RNA polymerase, sigma 32 (sigma H) factor; Sigma factors are initiation factors that promote the attachment of RNA polymerase to specific initiation sites and are then released, This sigma factor is responsible for the expression of heat shock promoters (284 aa) | oxaA | membrane protein insertase; Inner membrane protein required for the insertion of integral membrane proteins into the membrane, Probably plays an essential role in the integration of proteins of the respiratory chain complexes, Involved in integration of membrane proteins that insert dependently and independently of the Sec translocase complex, Essential for the integration of subunits a and c of the F(1)F(0)ATP synthase, and secE proteins (548 aa) | 199.34 | none | | |
| glpC | sn-glycerol-3-phosphate dehydrogenase (anaerobic), small subunit; Electron transfer protein; may also function as the membrane anchor for the glpAB dimer (396 aa) | nrfC | formate-dependent nitrite reductase, 4Fe4S subunit; Probably involved in the transfer of electrons from the quinone pool to the type-c cytochromes (223 aa) | 128.35 | none | | Electron transfer proteins |

| | | | | | | | |
|---|---|---|---|---|---|---|---|
| fruA | fused fructose-specific PTS enzymes- IIBcomponent/IIC components; The phosphoenolpyruvate-dependent sugar phosphotransferase system (sugar PTS), a major carbohydrate active -transport system, catalyzes the phosphorylation of incoming sugar substrates concomitantly with their translocation across the cell membrane, This system is involved in fructose transport (563 aa) | mtlA | fused mannitol-specific PTS enzymes- IIA components/IIB components/IIC components; The phosphoenolpyruvate-dependent sugar phosphotransferase system (sugar PTS), a major carbohydrate active -transport system, catalyzes the phosphorylation of incoming sugar substrates concomitantly with their translocation across the cell membrane, This system is involved in mannitol transport (637 aa) | 101.74 | none | | sugar PTS fructose & mannitol transport |
| recC | exonuclease V (RecBCD complex), gamma chain; Exhibits a wide variety of catalytic activities including ATP-dependent exonuclease, ATP-stimulated endonuclease, ATP-dependent helicase and DNA-dependent ATPase activities (1122 aa) | secE | preprotein translocase membrane subunit; Essential for protein export (127 aa) | 82.86 | none | | |
| trpA | tryptophan synthase, alpha subunit; The alpha subunit is responsible for the aldol cleavage of indoleglycerol phosphate to indole and glyceraldehyde 3-phosphate (By similarity) (268 aa) | hisH | imidazole glycerol phosphate synthase, glutamine amidotransferase subunit with HisF; IGPS catalyzes the conversion of PRFAR and glutamine to IGP, AICAR and glutamate, The hisH subunit provides the glutamine amidotransferase activity that produces the ammonia necessary to hisF for the synthesis of IGP and AICAR (196 aa) | 50.10 | Neighborhood in the Genome (homologous genes in other genomes): 0.788 Cooccurence: 0.303 Co-Mentioned in PubMed Abstracts (putative homologs mentioned together in other species): 0.227 | 0.87 | |

| | | | | | | |
|---|---|---|---|---|---|---|
| ihfB | integration host factor (IHF), DNA-binding protein, beta subunit; This protein is one of the two subunits of integration host factor, a specific DNA-binding protein that functions in genetic recombination as well as in transcriptional and translational control (By similarity) (94 aa) | rpoH | RNA polymerase, sigma 32 (sigma H) factor; Sigma factors are initiation factors that promote the attachment of RNA polymerase to specific initiation sites and are then released, This sigma factor is responsible for the expression of heat shock promoters (284 aa) | 44.51 | none | |
| trpA | tryptophan synthase, alpha subunit; The alpha subunit is responsible for the aldol cleavage of indoleglycerol phosphate to indole and glyceraldehyde 3-phosphate (By similarity) (268 aa) | trpE | component I of anthranilate synthase (520 aa) | 32.44 | Neighborhood in the Genome: 0.800 Cooccurence Across Genomes: 0.766 Co-Expression: 0.999 Co-Mentioned in PubMed Abstracts: 0.733 | 0.999 |
| trpA | tryptophan synthase, alpha subunit; The alpha subunit is responsible for the aldol cleavage of indoleglycerol phosphate to indole and glyceraldehyde 3-phosphate (By similarity) (268 aa) | hisF | imidazole glycerol phosphate synthase, catalytic subunit with HisH; IGPS catalyzes the conversion of PRFAR and glutamine to IGP, AICAR and glutamate, The hisF subunit catalyzes the cyclization activity that produces IGP and AICAR from PRFAR using the ammonia provided by the hisH subunit (By similarity) (258 aa) | 31.42 | Neighborhood in the Genome (homologous genes are neighbors in other genomes): 0.680 Co-Mentioned in PubMed Abstracts (putative homologs mentioned together in other species): 0.364 | 0.783 |

| | | | | | | |
|---|---|---|---|---|---|---|
| clpP | proteolytic subunit of ClpA-ClpP and ClpX-ClpP ATP-dependent serine proteases; Cleaves peptides in various proteins in a process that requires ATP hydrolysis, Has a chymotrypsin-like activity, Plays a major role in the degradation of misfolded proteins, May play the role of a master protease which is attracted to different substrates by different specificity factors such as clpA or clpX (207 aa) | pnp | polynucleotide phosphorylase/polyadenylase; Involved in mRNA degradation, Hydrolyzes single-stranded polyribonucleotides processively in the 3'- to 5'-direction (711 aa) | 15.06 | none | |
| tolB | periplasmic protein; Involved in the tonB-independent uptake of group A colicins (colicins A, E1, E2, E3 and K), Necessary for the colicins to reach their respective targets after initial binding to the bacteria (430 aa) | oxaA | membrane protein insertase; Inner membrane protein required for the insertion of integral membrane proteins into the membrane, Probably plays an essential role in the integration of proteins of the respiratory chain complexes, Involved in integration of membrane proteins that insert dependently and independently of the Sec translocase complex, Essential for the integration of subunits a and c of the F(1)F(0)ATP synthase, and secE proteins (548 aa) | 12.28 | none | Linked to transmembrane activity |

| | | | | | | | |
|---|---|---|---|---|---|---|
| clpX | ATPase and specificity subunit of ClpX-ClpP ATP-dependent serine protease; ATP-dependent specificity component of the Clp protease, It directs the protease to specific substrates, It may bind to the lambda O substrate protein and present it to the clpP protease in a form that can be recognized and readily hydrolyzed by clpP, Can perform chaperone functions in the absence of clpP (424 aa) | pnp | polynucleotide phosphorylase/polyadenylase; Involved in mRNA degradation, Hydrolyzes single-stranded polyribonucleotides processively in the 3'- to 5'-direction (711 aa) | 10.71 | none | | |
| recO | gap repair protein; Involved in DNA repair and recF pathway recombination (By similarity) (242 aa) | recC | exonuclease V (RecBCD complex), gamma chain; Exhibits a wide variety of catalytic activities including ATP-dependent exonuclease, ATP-stimulated endonuclease, ATP-dependent helicase and DNA-dependent ATPase activities (1122 aa) | 9.32 | Co-Mentioned in PubMed Abstracts: 0.741 | 0.74 | |
| crr | glucose-specific enzyme IIA component of PTS; The phosphoenolpyruvate-dependent sugar phosphotransferase system (sugar PTS), a major carbohydrate active - transport system, catalyzes the phosphorylation of incoming sugar substrates concomitantly with their translocation across the cell membrane, This system is involved in glucose transport (169 aa) | chbA | N,N'-diacetylchitobiose-specific enzyme IIA component of PTS; The phosphoenolpyruvate-dependent sugar phosphotransferase system (PTS), a major carbohydrate active - transport system, catalyzes the phosphorylation of incoming sugar substrates concomitant with their translocation across the cell membrane, This system is involved in N,N'-diacetylchitobiose transport (116 aa) | 7.73 | none | | sugar PTS glucose & N,N'-diacetylchitobiose transport |

| | | | | | | |
|---|---|---|---|---|---|---|
| acrB | multidrug efflux system protein; AcrAB is a drug efflux protein with a broad substrate specificity (1049 aa) | cusC | copper/silver efflux system, outer membrane component; Part of a cation efflux system that mediates resistance to copper and silver, In pathogenic strains it allows the bacteria to invade brain microvascular endothelial cells (BMEC) thus allowing it to cross the blood-brain barrier and cause neonatal meningitis (457 aa) | 6.94 | Neighborhood in the Genome (homologous genes are neighbors in other genomes): 0.716 Gene Fusions: 0.026 Cooccurence Across Genomes: 0.582 Co-Mentioned in PubMed Abstracts (putative homologs mentioned together in other species): 0.219 | 0.894 |
| oppD | oligopeptide transporter subunit; Part of the binding-protein-dependent transport system for oligopeptides, Probably responsible for energy coupling to the transport system (337 aa) | dppF | dipeptide transporter; Part of the binding-protein-dependent transport system for dipeptides, Probably responsible for energy coupling to the transport system (334 aa) | 6.28 | Neighborhood in the Genome (homologous genes are neighbors in other genomes): 0.310 Cooccurence Across Genomes: 0.766 Co-Expression: 0.575 | 0.724 |
| dnaK | chaperone HSP70. co-chaperone with DnaJ; Plays an essential role in the initiation of phage lambda DNA replication, where it acts in an ATP-dependent fashion with the dnaJ protein to release lambda O and P proteins from the preprimosomal complex, DnaK is also involved in chromosomal DNA replication, possibly through an analogous interaction with the dnaA protein, Also participates actively in the response to hyperosmotic shock (638 aa) | gyrA | DNA gyrase (type II topoisomerase), subunit A; DNA gyrase negatively supercoils closed circular double- stranded DNA in an ATP-dependent manner and also catalyzes the interconversion of other topological isomers of double-stranded DNA rings, including catenanes and knotted rings (875 aa) | 5.74 | Neighborhood in the Genome (homologous genes are neighbors in other genomes): 0.139 Cooccurence Across Genomes: 0.316 Experimental/Biochemical Data: 0.853 Co-Mentioned in PubMed Abstracts (putative homologs mentioned together in other species): 0.054 | 0.901 |

| | | | | | | | |
|---|---|---|---|---|---|---|---|
| aceF | pyruvate dehydrogenase, dihydrolipoyltransacetylase component E2; The pyruvate dehydrogenase complex catalyzes the overall conversion of pyruvate to acetyl-CoA and CO(2), It contains multiple copies of three enzymatic components- pyruvate dehydrogenase (E1), dihydrolipoamide acetyltransferase (E2) and lipoamide dehydrogenase (E3) (630 aa) | recO | gap repair protein; Involved in DNA repair and recF pathway recombination (By similarity) (242 aa) | 5.19 | none | | |
| chbA | N,N'-diacetylchitobiose-specific enzyme IIA component of PTS; The phosphoenolpyruvate-dependent sugar phosphotransferase system (PTS), a major carbohydrate active - transport system, catalyzes the phosphorylation of incoming sugar substrates concomitant with their translocation across the cell membrane, This system is involved in N,N'-diacetylchitobiose transport (116 aa) | mtlA | fused mannitol-specific PTS enzymes- IIA components/IIB components/IIC components; The phosphoenolpyruvate-dependent sugar phosphotransferase system (sugar PTS), a major carbohydrate active - transport system, catalyzes the phosphorylation of incoming sugar substrates concomitantly with their translocation across the cell membrane, This system is involved in mannitol transport (637 aa) | 4.56 | none | | sugar PTS N,N'-diacetylchitobiose & mannitol transport |
| trpE | component I of anthranilate synthase (520 aa) | hisF | imidazole glycerol phosphate synthase, catalytic subunit with HisH; IGPS catalyzes the conversion of PRFAR and glutamine to IGP, AICAR and glutamate, The hisF subunit catalyzes the cyclization activity that produces IGP and AICAR from PRFAR using the ammonia provided by the hisH subunit (By similarity) (258 aa) | 3.83 | Neighborhood in the Genome (homologous genes are neighbors in other genomes): 0.465 Gene Fusions: 0.322 Co-Expression (putative homologs are coexpressed in other species ): 0.112 | 0.633 | |

| | | | | | | |
|---|---|---|---|---|---|---|
| fliM | flagellar motor switching and energizing component; FliM is one of three proteins (fliG, fliN, fliM) that form a switch complex that is proposed to be located at the base of the basal body, This complex interacts with the cheY and cheZ chemotaxis proteins, in addition to contacting components of the motor that determine the direction of flagellar rotation | cheA | fused chemotactic sensory histidine kinase in two-component regulatory system with CheB and CheY: sensory histidine kinase/signal sensing protein; Involved in the transmission of sensory signals from the chemoreceptors to the flagellar motors, CheA is autophosphorylated; it can transfer its phosphate group to either cheB or cheY | 3.82 | Neighborhood in the Genome (homologous genes are neighbors in other genomes): 0.792 Cooccurence Across Genomes: 0.277 Co-Mentioned in PubMed Abstracts: 0.727 | 0.968 |
| trpA | tryptophan synthase, alpha subunit; The alpha subunit is responsible for the aldol cleavage of indoleglycerol phosphate to indole and glyceraldehyde 3-phosphate (By similarity) | leuC | 3-isopropylmalate dehydratase large subunit (EC:4,2,1,33); Catalyzes the isomerization between 2-isopropylmalate and 3-isopropylmalate, via the formation of 2-isopropylmaleate | 2.95 | Neighborhood in the Genome (homologous genes are neighbors in other genomes): 0.465 | 0.465 |
| ilvH | acetolactate synthase III, thiamin-dependent, small subunit (163 aa) | trpA | tryptophan synthase, alpha subunit; The alpha subunit is responsible for the aldol cleavage of indoleglycerol phosphate to indole and glyceraldehyde 3-phosphate (By similarity) (268 aa) | 2.60 | | |
| fliA | RNA polymerase, sigma 28 (sigma F) factor; Sigma factors are initiation factors that promote the attachment of RNA polymerase to specific initiation sites and are then released, This alternative sigma factor is specific for class 3 flagellar operons | flgG | flagellar component of cell-distal portion of basal-body rod | 2.46 | Neighborhood in the Genome (Neighborhood in the Genome): 0.788 Cooccurence Across Genomes: 0.577 Co-Expression: 0.778 Co-Mentioned in PubMed Abstracts: 0.606 | 0.991 |

| | | | | | | |
|---|---|---|---|---|---|---|
| cheW | purine-binding chemotaxis protein; Involved in the transmission of sensory signals from the chemoreceptors to the flagellar motors, It physically bridges cheA to the MCPs (methyl-accepting chemotaxis proteins) to allow regulated phosphotransfer to cheY and cheB (167 aa) | flgB | flagellar component of cell-proximal portion of basal-body rod (138 aa) | 2.39 | Neighborhood in the Genome (homologous genes are neighbors in other genomes): 0.588 Co-Expression: 0.521 | 0.789 |
| cheW | purine-binding chemotaxis protein; Involved in the transmission of sensory signals from the chemoreceptors to the flagellar motors, It physically bridges cheA to the MCPs (methyl-accepting chemotaxis proteins) to allow regulated phosphotransfer to cheY and cheB (167 aa) | flgG | flagellar component of cell-distal portion of basal-body rod (260 aa) | 2.35 | Neighborhood in the Genome (homologous genes are neighbors in other genomes): 0.719 Co-Expression: 0.219 | 0.765 |
| fliM | flagellar motor switching and energizing component; FliM is one of three proteins (fliG, fliN, fliM) that form a switch complex that is proposed to be located at the base of the basal body, This complex interacts with the cheY and cheZ chemotaxis proteins, in addition to contacting components of the motor that determine the direction of flagellar rotation (334 aa) | fliA | RNA polymerase, sigma 28 (sigma F) factor; Sigma factors are initiation factors that promote the attachment of RNA polymerase to specific initiation sites and are then released, This alternative sigma factor is specific for class 3 flagellar operons (239 aa) | 2.35 | Neighborhood in the Genome (homologous genes are neighbors in other genomes): 0.796 Cooccurence Across Genomes: 0.705 Co-Expression: 0.158 Co-Mentioned in PubMed Abstracts: 0.697 | 0.983 |

| | | | | | |
|---|---|---|---|---|---|
| secB | protein export chaperone; One of the proteins required for the normal export of preproteins out of the cell cytoplasm, It is a molecular chaperone that binds to a subset of precursor proteins, maintaining them in a translocation-competent state, It also specifically binds to its receptor secA (155 aa) | parC | DNA topoisomerase IV, subunit A; Topoisomerase IV is essential for chromosome segregation, It has relaxation of supercoiled DNA activity, Performs the decatenation events required during the replication of a circular DNA molecule (752 aa) | 2.34 | |
| hflK | modulator for HflB protease specific for phage lambda CII repressor; HflC and hflK govern the stability of phage lambda cII protein and have been proposed to encode or regulate a cII- specific protease (419 aa) | ccmE | periplasmic heme chaperone; Heme chaperone required for the biogenesis of c-type cytochromes, Transiently binds heme delivered by ccmC and transfers the heme to apocytochromes in a process facilitated by ccmF and ccmH (159 aa) | 1.98 | |
| malE | N,N'-diacetylchitobiose-specific enzyme IIA component of PTS; The phosphoenolpyruvate-dependent sugar phosphotransferase system (PTS), a major carbohydrate active -transport system, catalyzes the phosphorylation of incoming sugar substrates concomitant with their translocation across the cell membrane, This system is involved in N,N'-diacetylchitobiose transport (116 aa) | chbA | N,N'-diacetylchitobiose-specific enzyme IIA component of PTS; The phosphoenolpyruvate-dependent sugar phosphotransferase system (PTS), a major carbohydrate active -transport system, catalyzes the phosphorylation of incoming sugar substrates concomitant with their translocation across the cell membrane, This system is involved in N,N'-diacetylchitobiose transport (116 aa) | 1.91 | sugar PTS N,N'-diacetylchitobiose transport |

| | | | | | | |
|---|---|---|---|---|---|---|
| ruvA | component of RuvABC resolvasome, regulatory subunit; The ruvA-ruvB complex in the presence of ATP renatures cruciform structure in supercoiled DNA with palindromic sequence, indicating that it may promote strand exchange reactions in homologous recombination, RuvAB is an helicase that mediates the Holliday junction migration by localized denaturation and reannealing, RuvA stimulates, in the presence of DNA, the weak ATPase activity of ruvB, Binds both single- and double-stranded DNA (dsDNA), Binds preferentially to supercoiled rather than to relaxed dsDNA | recR | gap repair protein; May play a role in DNA repair, It seems to be involved in an recBC-independent recombinational process of DNA repair, It may act with recF and recO | 1.84 | Neighborhood in the Genome (homologous genes are neighbors in other genomes): 0.343 Cooccurence Across Genomes: 0.192 Co-Mentioned in PubMed Abstracts: 0.662 | 0.873 |
| secE | preprotein translocase membrane subunit; Essential for protein export (127 aa) | tolB | periplasmic protein; Involved in the tonB-independent uptake of group A colicins (colicins A, E1, E2, E3 and K), Necessary for the colicins to reach their respective targets after initial binding to the bacteria (430 aa) | 1.45 | | |
| secB | protein export chaperone; One of the proteins required for the normal export of preproteins out of the cell cytoplasm, It is a molecular chaperone that binds to a subset of precursor proteins, maintaining them in a translocation-competent state, It also specifically binds to its receptor secA (155 aa) | tolB | periplasmic protein; Involved in the tonB-independent uptake of group A colicins (colicins A, E1, E2, E3 and K), Necessary for the colicins to reach their respective targets after initial binding to the bacteria (430 aa) | 1.05 | | |

| | | | | |
|---|---|---|---|---|
| rpoH | RNA polymerase, sigma 32 (sigma H) factor; Sigma factors are initiation factors that promote the attachment of RNA polymerase to specific initiation sites and are then released, This sigma factor is responsible for the expression of heat shock promoters (284 aa) | flgF | flagellar component of cell-proximal portion of basal-body rod (251 aa) | 0.84 |
| recO | gap repair protein; Involved in DNA repair and recF pathway recombination (By similarity) (242 aa) | secG | preprotein translocase membrane subunit; Involved in protein export, Participates in an early event of protein translocation (110 aa) | 0.81 |
| recO | gap repair protein; Involved in DNA repair and recF pathway recombination (By similarity) (242 aa) | rne | fused ribonucleaseE-endoribonuclease/RNA-binding protein/RNA degradosome binding protein; Matures 5S rRNA from its precursors from all the rRNA genes, It also cleaves RNA I, a molecule that controls the replication of colE1 plasmid DNA, It is the major endoribonuclease participating in mRNA turnover in E,coli, It initiates the decay of RNAs by cutting them internally near their 5'-end, It is able to remove poly(A) tails by an endonucleolytic process (1061 aa) | 0.79 |

| | | | | | | |
|---|---|---|---|---|---|---|
| rpoH | | rne | fused ribonucleaseE-endoribonuclease/RNA-binding protein/RNA degradosome binding protein; Matures 5S rRNA from its precursors from all the rRNA genes, It also cleaves RNA I, a molecule that controls the replication of colE1 plasmid DNA, It is the major endoribonuclease participating in mRNA turnover in E,coli, It initiates the decay of RNAs by cutting them internally near their 5'-end, It is able to remove poly(A) tails by an endonucleolytic process (1061 aa) | 0.65 | | |
| fliA | RNA polymerase, sigma 28 (sigma F) factor; Sigma factors are initiation factors that promote the attachment of RNA polymerase to specific initiation sites and are then released, This alternative sigma factor is specific for class 3 flagellar operons (239 aa) | flgC | flagellar component of cell-proximal portion of basal-body rod (134 aa) | 0.59 | Neighborhood in the Genome (homologous genes are neighbors in other genomes): 0.777 Co-Expression: 0.864 Experimental/Biochemical Data: 0.496 Co-Mentioned in PubMed Abstracts: 0.723 | 0.994 |

| | | | | | | | |
|---|---|---|---|---|---|---|---|
| ruvB | ATP-dependent DNA helicase, component of RuvABC resolvasome; The ruvA-ruvB complex in the presence of ATP renatures cruciform structure in supercoiled DNA with palindromic sequence, indicating that it may promote strand exchange reactions in homologous recombination, RuvAB is an helicase that mediates the Holliday junction migration by localized denaturation and reannealing (336 aa) | recR | gap repair protein; May play a role in DNA repair, It seems to be involved in an recBC-independent recombinational process of DNA repair, It may act with recF and recO (201 aa) | 0.55 | Neighborhood in the Genome (homologous genes are neighbors in other genomes): 0.513 Cooccurence Across Genomes: 0.405 Co-Mentioned in PubMed Abstracts: 0.667 | 0.89 | |
| mtlA | fused mannitol-specific PTS enzymes- IIA components/IIB components/IIC components; The phosphoenolpyruvate-dependent sugar phosphotransferase system (sugar PTS), a major carbohydrate active - transport system, catalyzes the phosphorylation of incoming sugar substrates concomitantly with their translocation across the cell membrane, This system is involved in mannitol transport (637 aa) | crr | glucose-specific enzyme IIA component of PTS; The phosphoenolpyruvate-dependent sugar phosphotransferase system (sugar PTS), a major carbohydrate active - transport system, catalyzes the phosphorylation of incoming sugar substrates concomitantly with their translocation across the cell membrane, This system is involved in glucose transport (169 aa) | 0.47 | | | PTS sugar Mannitol and glucose transport |
| dnaQ | DNA polymerase III epsilon subunit; DNA polymerase III is a complex, multichain enzyme responsible for most of the replicative synthesis in bacteria, The epsilon subunit contain the editing function and is a proofreading 3'-5' exonuclease (243 aa) | secB | protein export chaperone; One of the proteins required for the normal export of preproteins out of the cell cytoplasm, It is a molecular chaperone that binds to a subset of precursor proteins, maintaining them in a translocation-competent state, It also specifically binds to its receptor secA (155 aa) | 0.44 | Neighborhood in the Genome (homologous genes are neighbors in other genomes): 0.439 Cooccurence Across Genomes: 0.202 | 0.522 | |

| | | | | | | |
|---|---|---|---|---|---|---|
| trpB | tryptophan synthase, beta subunit; The beta subunit is responsible for the synthesis of L-tryptophan from indole and L-serine (By similarity) (397 aa) | leuC | 3-isopropylmalate dehydratase large subunit (EC-4,2,1,33); Catalyzes the isomerization between 2-isopropylmalate and 3-isopropylmalate, via the formation of 2-isopropylmaleate (466 aa) | 0.36 | Neighborhood in the Genome (homologous genes are neighbors in other genomes): 0.412 | 0.412 |
| cysA | sulfate/thiosulfate transporter subunit; Part of the ABC transporter complex cysAWTP involved in sulfate/thiosulfate import, Responsible for energy coupling to the transport system (365 aa) | nuoL | NADH-ubiquinone oxidoreductase, membrane subunit L; NDH-1 shuttles electrons from NADH, via FMN and iron- sulfur (Fe-S) centers, to quinones in the respiratory chain, The immediate electron acceptor for the enzyme in this species is believed to be ubiquinone, Couples the redox reaction to proton translocation (for every two electrons transferred, four hydrogen ions are translocated across the cytoplasmic membrane), and thus conserves the redox energy in a proton gradient (613 aa) | 0.34 | | |

| | | | | | | |
|---|---|---|---|---|---|---|
| ihfA | integration host factor (IHF), DNA-binding protein, alpha subunit; This protein is one of the two subunits of integration host factor, a specific DNA-binding protein that functions in genetic recombination as well as in transcriptional and translational control (By similarity) (99 aa) | rne | fused ribonucleaseE-endoribonuclease/RNA-binding protein/RNA degradosome binding protein; Matures 5S rRNA from its precursors from all the rRNA genes, It also cleaves RNA I, a molecule that controls the replication of colE1 plasmid DNA, It is the major endoribonuclease participating in mRNA turnover in E,coli, It initiates the decay of RNAs by cutting them internally near their 5'-end, It is able to remove poly(A) tails by an endonucleolytic process (1061 aa) | 0.31 | | |
| dnaJ | chaperone HSP40. co-chaperone with DnaK; Interacts with dnaK and grpE to disassemble a protein complex at the origins of replication of phage lambda and several plasmids, Participates actively in the response to hyperosmotic and heat shock by preventing the aggregation of stress-denatured proteins and by disaggregating proteins, also in an autonomous, dnaK-independent fashion, Unfolded proteins bind initially to dnaJ; upon interaction with the dnaJ-bound protein, dnaK hydrolyzes its bound ATP, resulting in the formation of a stable complex, GrpE releases ADP from dnaK; ATP binding to d [,,,] (376 aa) | gyrA | DNA gyrase (type II topoisomerase), subunit A; DNA gyrase negatively supercoils closed circular double- stranded DNA in an ATP-dependent manner and also catalyzes the interconversion of other topological isomers of double-stranded DNA rings, including catenanes and knotted rings (875 aa) | 0.28 | Neighborhood in the Genome (homologous genes are neighbors in other genomes): 0.247 Cooccurence Across Genomes: 0.363 Experimental/Biochemical Data: 0.371 | 0.656 |

| | | | | | | |
|---|---|---|---|---|---|---|
| secB | protein export chaperone; One of the proteins required for the normal export of preproteins out of the cell cytoplasm, It is a molecular chaperone that binds to a subset of precursor proteins, maintaining them in a translocation-competent state, It also specifically binds to its receptor secA (155 aa) | ompW | outer membrane protein W; Acts as a receptor for colicin S4 (212 aa) | 0.25 | | |
| pnp | polynucleotide phosphorylase/polyadenylase; Involved in mRNA degradation, Hydrolyzes single-stranded polyribonucleotides processively in the 3'- to 5'-direction (711 aa) | ruvC | component of RuvABC resolvasome, endonuclease; Nuclease that resolves Holliday junction intermediates in genetic recombination, Cleaves the cruciform structure in supercoiled DNA by nicking to strands with the same polarity at sites symmetrically opposed at the junction in the homologous arms and leaves a 5'-terminal phosphate and a 3'-terminal hydroxyl group (173 aa) | 0.22 | | |
| atpG | F1 sector of membrane-bound ATP synthase, gamma subunit; Produces ATP from ADP in the presence of a proton gradient across the membrane, The gamma chain is believed to be important in regulating ATPase activity and the flow of protons through the CF(0) complex (By similarity) (287 aa) | glyQ | glycine tRNA synthetase, alpha subunit (303 aa) | 0.22 | Neighborhood in the Genome (homologous genes are neighbors in other genomes): 0.323 Co-Expression (putative homologs coexpressed in other species): 0.265 | 0.468 |

| | | | | | | |
|---|---|---|---|---|---|---|
| rpoD | RNA polymerase, sigma 70 (sigma D) factor; Sigma factors are initiation factors that promote the attachment of RNA polymerase to specific initiation sites and are then released, This is the primary sigma factor of this bacterium | rpoH | RNA polymerase, sigma 32 (sigma H) factor; Sigma factors are initiation factors that promote the attachment of RNA polymerase to specific initiation sites and are then released, This sigma factor is responsible for the expression of heat shock promoters | 0.21 | Neighborhood in the Genome (homologous genes are neighbors in other genomes): 0.124 Cooccurence Across Genomes: 0.766 Co-Mentioned in PubMed Abstracts: 0.951 | 0.546 |
